# DWI-guided DP-IMRT and conventional MRI-based IMRT in head and neck squamous cell carcinoma: a comparative study

**DOI:** 10.1186/s12885-025-14684-x

**Published:** 2025-08-23

**Authors:** Chao Tan, Yuyi Li, Cuihong Jiang, Lili He, Shuai Xiao, Changgen Fan, Xu Ye, Qi Zhao, Wenqiong Wu, Yanxian Li, Feng Liu

**Affiliations:** 1 The Affiliated Cancer Hospital of Xiangya School of Medicine, Hunan Cancer Hospital, Central South University, Changsha, Hunan China; 2 Graduate Collaborative Training Base of Hunan Cancer Hospital, Hengyang Medical School, University of South China, Hengyang, Hunan China

**Keywords:** Head and neck squamous cell carcinoma, Chemoradiotherapy, DWI imaging, Radiotherapy

## Abstract

**Background:**

The impact of diffusion-weighted magnetic resonance imaging guided dose-painting intensity-modulated radiation therapy on head and neck squamous cell carcinoma remains unclear. Our objective aimed at comparing the outcomes and toxicities of DWI-guided DP-IMRT for HNSCC.

**Methods and materials:**

Between June 2018 and November 2022, we conducted a retrospective analysis of 260 HNSCC patients. Among them, 126 received chemoradiotherapy using DWI-guided dose painting intensity-modulated radiation therapy (DWI group), and the remaining 134 underwent chemoradiotherapy using conventional MRI-based intensity-modulated radiation therapy (Standard group). In DWI group, DW-MRI-guided gross tumor volume (GTV) received escalation to 77 Gy/35 fx in 2.2 Gy per fraction, while in Standard group, the GTV of HNSCC was irradiated at 70 Gy/35 fx at 2.0 Gy per fraction. All patients received induction chemotherapy. Survival rates were compared, and multivariate analyses were conducted.

**Results:**

The median follow-up duration was 23 months. Compared to conventional MRI-based intensity-modulated radiation therapy, DWI-guided dose painting intensity-modulated radiation therapy showed improved 2-year disease-free survival (75.2% vs. 63.8%; *P* =.011) and locoregional recurrence-free survival (75.9% vs.64.7%; *P* =.013). Overall survival did not show significant difference between DWI group and Standard group (*P* =.442). Grade 3–5 toxicities were similar between the two groups. Multivariate analysis showed that DWI-guided dose painting was an independent prognostic factor for DFS (HR 0.559, 95% CI 0.324–0.966, *P* =.037).

**Conclusion:**

DWI-guided dose escalation has a benefit in DFS for patients with HNSCC without increasing acute adverse events.

## Introduction

Head and neck squamous cell carcinoma (HNSCC), arising from in the mucosal epithelium, are the most prevalent malignancies in the head and neck region [1], including oral cavity, larynx, oropharynx and hypopharynx. In 2018, HNSCC ranked as the seventh most common cancer globally [2], with its incidence projected to surge by 30% by 2030. Chemoradiotherapy (CCRT) stands as the standard treatment modality for unresected locally advanced HNSCC [3–7] (LA-HNSCC). Locoregional control failure is the primary cause of recurrence in patients with LA-HNSCC. Notably, in head and neck (HN) radiotherapy, heterogeneity in target volume selection significantly influences tumor variations [8]. Boosting radiation dose to the gross tumor volume (GTV) has demonstrated potential for enhancing local control (LC) [9–11]. However, escalated tumor radiation doses have yielded suboptimal outcomes in terms of both toxicity and efficacy [12, 13], necessitating a study of higher radiation doses tailored to optimal tumor volumes.

Magnetic resonance imaging (MRI)-guided radiotherapy has heralded a new era, offering superior soft tissue contrast and obviating ionizing radiation, rendering it preferable for imaging soft tissues like the brain, prostate, and liver. This affords more accurate identification and tracking of the target and organs at risk (OARs), which is crucial for delivering the correct radiation dose to the gross tumor meanwhile decreasing damage to normal organs by surrounding [14, 15]. One of promising techniques for dose painting (DP) is diffusion-weighted magnetic resonance imaging (DW-MRI), with several studies linking pretreatment DW-MRI to treatment outcomes in HNSCC [16, 17]. The apparent diffusion coefficient (ADC) targets of a tumor lesion have been shown to function as a marker for prediction of response to chemoradiation in NPC and other HNSCC. A retrospective study [16] demonstrated that DWI-guided dose escalation represents a practical way capable of enhancing local control in patients with locoregionally advanced nasopharyngeal carcinoma (LA-NPC), while being tolerable in terms of treatment-related complications.

To date, no clinical studies have juxtaposed DWI-guided dose-painting intensity-modulated radiation therapy (DP-IMRT) with conventional MRI-based IMRT for LA-HNSCC. Therefore, this retrospective study compared DP-IMRT with conventional MRI-based IMRT. We assumed that by DWI-guided DP, dose-escalation radiotherapy could be determined to the appropriate tumor volume for HNSCC. This improvement is expected to enhance efficacy without increasing treatment-related toxicity.

## Materials and methods

### Patients

**Fig. 1:**
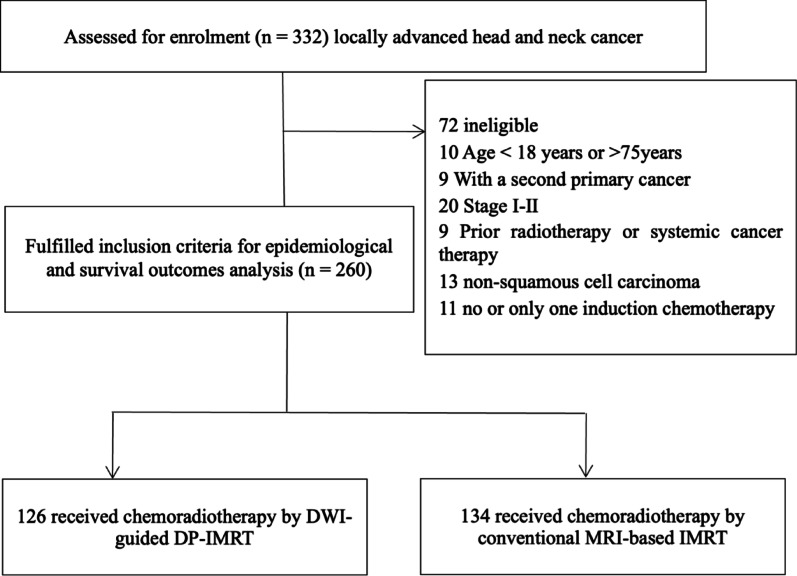
Flow chart demonstrating the study cohort

Between June 2018 and November 2022, we retrospectively analyzed 260 patients with nonoperative (unresectable or surgery-declined), locally advanced HNSCC from Hunan Cancer Hospital in this study (Fig. 1). Eligibility criteria comprised locally advanced head and neck squamous cell carcinoma (oral, oropharyngeal, hypopharyngeal, and laryngeal) confirmed by pathology or histology, except nasopharyngeal carcinoma; stage T3-4N0-3M0 (8th International Union Against Cancer); age between 18 and 75 years; normal function of major organs, as evidenced by routine blood and biochemical tests meeting standards; and a Karnofsky performance score of 70 or more. Exclusion criteria included prior radiotherapy, another malignancy, surgery, evidence of distant metastasis, uncontrolled severe illness, lactation and pregnancy. Among them, 126 received chemoradiotherapy using DWI-guided dose painting intensity-modulated radiation therapy (DWI group or group A), and the remaining 134 underwent chemoradiotherapy using conventional MRI-based intensity-modulated radiation therapy (Standard group or group B). The study protocol was approved by the Ethics Committee of the Hunan Cancer Hospital.

### Imaging

All patients underwent contrast-enhanced magnetic resonance imaging (MRI) of the head and neck using a 1.5-Tesla scanner (Magnetom Sonata; Siemens, Erlangen, Germany). Conventional MRI included T1- and T2-weighted imaging, T1-contrast-enhanced sequences, and DWI imaging. ADC values were automatically calculated by the operating console of the MR imaging device and were displayed as corresponding ADC maps. Computed tomography (CT) simulation images were fused with T1-weighted MR images in both groups. And in DWI group, DWI images were fused to the CT simulation images.

We used deformable registration of the DWI image (b = 0 s/mm^2^ image) to T1-weighted MRI to correct the distortion caused by sensitive artifacts [18]. Using a semiautomatic registration method can reduce the mean registration error between DWI and anatomical images [17].

### Target delineation

**Fig. 2:**
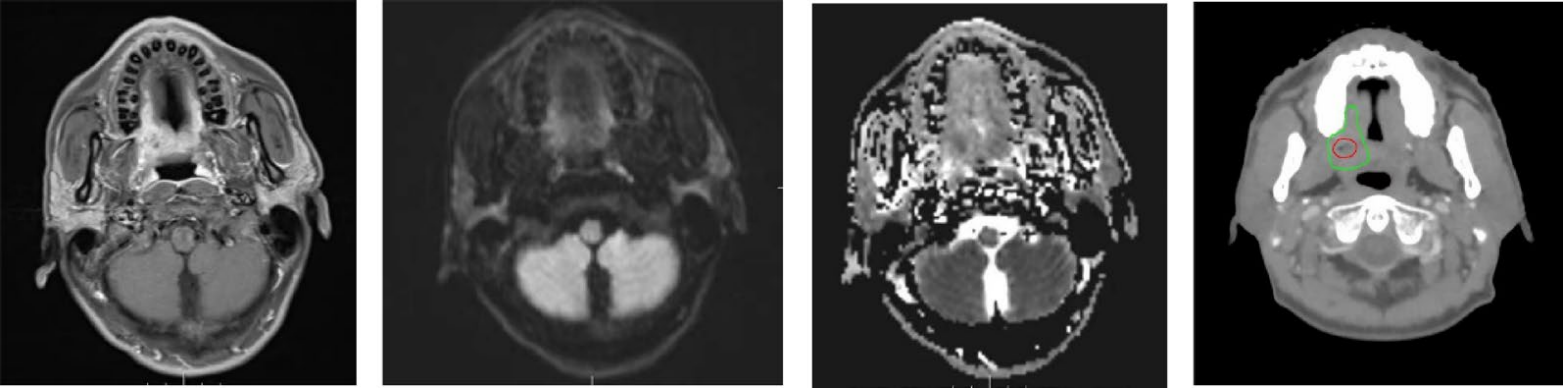
Example of target mapping of IMRT plan for a patient with cT2N2M0 oropharynx carcinoma by diffusion-weighted magnetic resonance imaging guided dose mapping before induction chemotherapy. (**A**) Contrast-enhanced T1-weighted sequence. (**B**) DWI sequence. (**C**) Apparent diffusion coefficient map. (**D**) Transverse slice of target volumes. Targets: high-dose gross target volume (GTVp-dwi) (red) and gross tumor volume (GTVp) (green) were delineated at apparent diffusion coefficient map fused with computed tomography simulation image

To capture both superficial and deep macroscopic tumor extensions, all patients underwent clinical examination, head and neck endoscopy and anatomic diagnostic imaging with CT and MRI. The gross tumor volume (GTVp) was defined based on fiberoptic nasopharyngoscopy, planning CT, MRI data. (Fig. 2). Dose painting by contours was employed to uniformly redistribute escalated doses to the DWI-guided primary gross tumor volume (GTV-dwi). The clinical target volume (CTV1) encompassed the GTVp with a 5 mm margin and the involved lymph nodes. CTV2 included adjacent suspected subclinical extensions of the GTVp. All the radiotherapy used conventional segmentation pattern, once a day, 5 times a week.

### DWI-guided DP

GTV delineation on DW-MRI was performed on an ADC map [19]. The mean ADC defined as reporting ADC values within a tumor region [20]. Values in the HN area has reported largely variation, ranging from 0.4 × 10^3^ mm^2^/s to 1.4 × 10^3^ mm^2^/s. Moreover, a low ADC indicates tumor presence and is associated with worse outcomes [21]. Thus, areas within the GTVp with an ADC below the mean ADC were segmented as GTVp-dwi according to the MRI before treatment. The planning target volume (PTV) was defined as a 5 mm expansion around CTV. GTV contours were manually delineated by two radiation oncologists specializing in the HN region.

All patients underwent intensity-modulated radiation therapy used a 6 MV photon beam and a linear accelerator. An external beam algorithm was used to calculate the planned dose. Standard radiation therapy dose was delivered with IMRT at 70 Gy (35 fractions) to the GTVp, 66 Gy/35 fractions to PTV1, and 54 Gy/35 fractions to PTV2. In the DWI group, the dose of GTV-dwi was increased to 77 Gy in 35 fractions. Dose limitation to the brain stem and spinal cord were 54 Gy and 45 Gy to Dmax, respectively. Both groups utilized the same predefined constraints for the OARs, according to National Comprehensive Cancer Network.

### Chemotherapy

The induction chemotherapy regimen in both groups was TPF (cisplatin 75 mg/m^2^ on day 1, docetaxel 75 mg/m^2^ on day 1, and 5-fluorouracil 750 mg/m^2^ on days 1–5) [22, 23]. And CCRT was cisplatin (100 mg/m^2^) every 3 weeks, concurrently with IMRT. The whole patients underwent 2–3 cycles of both induction and concurrent chemotherapy.

### Follow-up

Follow-up duration began on the day of treatment and ended on the late follow-up (November 1, 2022), or until death. All acute and late adverse events were evaluated based on the Common Terminology Criteria for Adverse Events (version 5.0) and the Radiation Therapy Oncology Group (RTOG) Acute and Late Radiation Morbidity Scoring Criteria [24]. Three months after completing treatment, all patients underwent evaluation for complete tumor response (CR) through head and neck physical examination, along with MRI. The Response Evaluation Criteria in Solid Tumors [25] was applied to evaluated tumor responses.

### Statistical analysis

The two primary endpoints were disease-free survival (DFS) and local recurrence-free survival (LRFS). DFS was determined from the date of diagnosis to tumor recurrence, metastasis, or death, whichever occurred first. LRFS was calculated from the date of diagnosis to the date of documented local recurrence or death from any cause. While secondary endpoints comprised regional recurrence-free survival (RRFS), locoregional recurrence-free survival (LRRFS), distant metastasis-free survival (DMFS) and overall survival (OS).

We employed the χ^2^ test to analyze classification variables. DFS, LRFS, RRFS, LRRFS, DMFS and OS were assessed using Kaplan-Meier survival curves and compared by log-rank tests between groups A and B. Additionally, Univariate and multivariate survival prognostic analyses was evaluated using the Cox proportional hazards model. The potentially significant prognostic factors considered in the model included DWI-guided DP (DWI-guided DP-IMRT vs. conventional MRI-based IMRT without DP), patients age (≥ 56 vs. < 56 years), sex (male vs. female), human papillomavirus (HPV) status (negative vs. positive), tumor stage (T1-2 vs. T3-4), node stage (N0-1 vs. N2-3), KPS (90–100 vs. 70–80), primary tumor site (oropharyngeal cancer and non- oropharyngeal cancer). All analyses were performed using Statistical Product and Service Solutions version 27.0 (IBM Corp., Armonk, NY, USA), with statistical significance set at a P value below 0.05.

## Results

### Patient characteristics

Groups A and B comprised 126 and 134 patients, respectively. The median patient age was 56 (18–75 years), and the median follow-up time was 23 months. The mean ADC was 1.17 × 10^3^ mm^2^/s in both groups before treatment. Table 1 outlines the baseline features of the patients. Between the two groups, no significant differences were observed regarding clinical features or baseline demographics.

**Table 1:** Baseline clinical characteristics

Characteristics	DWI-Guided DP-IMRT Number of patients (%)	Conventional MRI-based IMRT Number of patients (%)	*P* value
Total	126	134	
Age, y			0.412
Median	57	56	
Range	31–75	31–75	
Sex			0.166
Male	115 (91.3)	130 (97)	
Female	11 (8.7)	4 (3)	
Karnofsky Scale			0.502
90–100	123 (97.6)	128 (95.5)	
70–80	3 (2.4)	6 (4.5)	
Disease Primary Site			0.086
Oral Cavity	18 (14.3)	16 (11.9)	
Oropharynx	48 (38.1)	37 (27.6)	
Larynx	3 (2.4)	10 (7.5)	
Hypopharynx	57 (45.2)	71 (53.0)	
Disease State			0.832
III	21 (16.7)	20 (14.9)	
IV	105 (83.3)	114 (85.1)	
T Stage			0.652
T1	10 (7.9)	9 (6.7)	
T2	37 (29.4)	48 (35.9)	
T3	24 (19.0)	20 (14.9)	
T4	55 (43.7)	57 (42.5)	
N Stage			0.918
N0	6 (4.8)	8 (5.9)	
N1	25 (19.8)	23 (17.2)	
N2	80 (63.5)	88 (65.7)	
N3	15 (11.9)	15 (11.2)	
p16			0.137
Positive	13 (10.3)	8 (6.0)	
Negative	113 (89.7)	126 (94.0)	
Immunotherapy	35 (27.8)	24 (14.3)	

### Response

The objective response rates (ORRs) were 100% in both groups. However, the CR rates differed significantly between DWI group and Standard group, with rates of 88.9% and 77.6%, respectively (*P* =.015). 14 patients in DWI group and 30 patients in Standard group had occurred partial response (PR). Additionally, salvage surgery was implemented on 18 patients with residual tumors (6 in DWI group and 12 in Standard group). Six months after the completion of CCRT, seven patients with residual neck lymph nodes (2 in DWI group and 5 in Standard group) underwent successful salvage neck dissection.

### Treatment compliance

The details of treatment compliance are provided in Table 2. In DWI group, 117 patients (92.9%) completed two cycles of induction chemotherapy, while 127 patients (94.8%) in Standard group did so. Suspension of the third cycle of induction chemotherapy occurred due to grade 4 oral mucositis and mucosal infection. During radiotherapy, 103 patients (81.7%) in DWI group received three cycles of cisplatin. Cumulative cisplatin doses ≥ 200 mg/m^2^ were administered to 117 patients (92.8%) patients and 127 patients (94.8%) in DWI group and Standard group, respectively. Cisplatin discontinuation due to acute adverse events occurred in 29 patients (21.6%), all during CCRT. Notably, only 50% of patients achieved the targeted three high-dose cisplatin applications during conventional radiotherapy. Furthermore, five patients did not receive the full planned dose of radiotherapy due to patient refusal and severe hematological toxicity. Importantly, none of the patients experienced treatment delays exceeding 5 days.

**Table 2:** Treatment compliance details

Treatment	No. (%)		
	DWI group (*n* = 126)	Standard group (*n* = 134)	All Patients (*n* = 260)
Cisplatin 100 mg/m^2^ IV Q3W			
Cycle 1	9 (7.1)	7 (5.2)	16 (6.2)
Cycle 2	14 (11.1)	22 (16.4)	36 (13.8)
Cycle 3	103 (81.7)	105 (78.4)	208 (80.0)
RT			
RT completed without interruption	2 (1.6)	3 (2.2)	5 (1.9)
Treatment delay, days			
>5	0 (0)	0 (0)	0 (0)
≦ 5	2 (1.6)	3 (2.2)	5 (1.9)
RT completed as prescribed dose	124 (98.4)	131 (97.8)	255 (98.0)
Duration, days			
Mean (range)	46.5 (43–50)	47 (44–50)	46.5 (43–50)
Smoking			
Yes	115 (91.3)	127 (94.8)	242 (93.1)
No	11 (8.7)	7 (5.2)	18 (6.9)

### Adverse events

All adverse events are listed in Table 3. The two groups showed no differences in toxicity. However, two patients developed acute liver dysfunction, which was considered to be cisplatin-related. Additionally, one patient in DWI group developed a pharyngoesophageal stricture due to the lower pharyngeal constrictors and cervical esophageal inlet was received a high dose. Dysphagia and mucositis were the most common grade 3–4 acute adverse events in this study. Fortunately, no grade 5 toxicity (death) happened both the two groups.

**Table 3:** Grade 3 to 4 toxicity

	DWI group	Standard group	
Adverse events	No. of patients (%)	No. of patients (%)	P value
Radiation dermatitis	15 (11.9)	11 (8.2)	0.321
Anemia	8 (6.3)	10 (7.0)	0.724
Neutropenia	12 (9.5)	18 (13.4)	0.324
Leukopenia	8 (6.3)	6 (6.7)	0.504
Lymphocyte count decreased	95 (75.4)	113 (84.3)	0.072
Hyponatremia	1 (0.8)	0	0.485
Hypomagnesemia	0	0	-
Hypokalemia	1 (0.8)	0	0.485
Thrombocytopenia	3 (2.4)	1 (0.7)	0.357
Liver dysfunction	2 (1.6)	0	0.234
Nephrotoxicity	0	0	-
Fatigue	23 (18.3)	16 (11.9)	0.154
Nausea	55 (43.7)	61 (45.4)	0.762
Vomiting	30 (23.8)	23 (17.2)	0.184
Grade 3 oral mucositis	75 (59.5%)	89 (66.4)	0.250
Grade 4 oral mucositis	2 (2.6%)	3 (2.2%)	1.000
Hypothyroidism	0	0	-
Sepsis	0	0	-
Sore throat	41 (32.5)	31 (23.1)	0.090
Constipation	0	0	-
Hoarseness	0	0	-
Late adverse events			
Lymphedema	0	0	-
Esophagitis	1 (0.8)	1 (0.7)	1.000
Diarrhea	0	0	-
Dysphagia	53 (42.1)	62 (42.3)	0.495
Dry mouth	15 (11.9)	10 (7.5)	0.225
Ototoxicity	0	0	-
Weight loss	11 (8.7)	14 (10.4)	0.639

### Treatment failure

The median follow-up time was 23 months (range: 9–62 months). In DWI group, 23 patients (18.3%) experienced locoregional tumor recurrence, while in Standard group, 40 patients (29.9%) faced the same. Among these cases, one patient in DWI group and five in Standard group experienced local-only failure, while 19 patients in DWI group and 34 patients in Standard group also experienced distant metastases. Of the 53 patients with distant organ metastases, 13 had liver, 13 had bone, and 27 had lung, with ten patients having metastases in more than one organ. Patients with relapse received salvage treatments.

### Survival

**Fig. 3:**
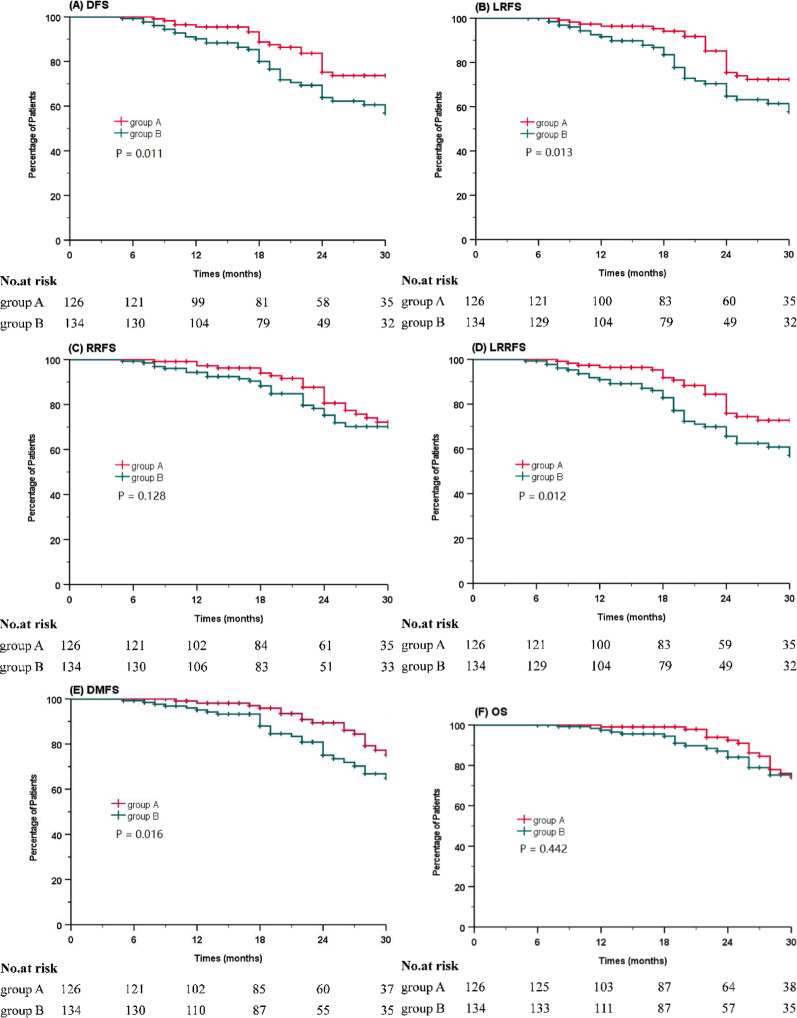
Kaplan-Meier survival curves for DWI-guided DP-IMRT and conventional MRI-based IMRT without DP groups. DFS **(A)**, LRFS **(B)**, RRFS **(C)**, LRRFS **(D)**, DMFS **(E)**, and OS **(F)**

A total of 39 deaths were reported, with 18 occurring in DWI group and 21 in Standard group. In DWI group, deaths were attributed to multiple distant recurrences (10 patients), severe malnutrition (6 patients), and non-radiation-induced cerebral hemorrhage (2 patients). In contrast, deaths in Standard group were mainly due to distant metastases (12 patients), local recurrence, severe malnutrition (7 patients), cardio-cerebrovascular events (1 patient), and traumatic brain injury (1 patient). The 2-year DFS rates were 75.2% and 63.8% in Groups DWI and Standard, respectively (*P* =.011), while the 2-year LRFS rates were 75.4% and 64.7% in Groups DWI and Standard, respectively (*P* =.013). The 2-year RRFS rates were 80.6% and 75.2% in Groups DWI and Standard, respectively (*P* =.128). The 2-year LRRFS rates were 75.9% and 65.7% in Groups DWI and Standard, respectively (*P* =.012). Similarly, the 2-year DMFS rates was 84.5% and 76.0% in groups DWI and Standard, respectively (*P* =.016), and the 2-year OS rates was 92.5% and 84.1% in groups DWI and Standard, respectively (*P* =.442). Notably, DWI group showed significantly higher 2-year DFS, LRFS, LRRFS, and DMFS rates compared to conventional MRI group (Fig. 3).

### Prognostic factors

Univariate analysis identified DWI-guided DP as a significant prognostic factor for 2-year DFS and LRFS (*P* =.011, *P* =.013, respectively). The T-category was also recognized as a prognostic factor for DFS, LRFS, RRFS, LRRFS, and OS. Additionally, the Karnofsky scale score showed a correlation between DFS and LRFS. However, age and primary tumor site did not emerge as a significant factor for DFS, LRFS, RRFS, LRRFS, DMFS, or OS. Multivariate analyses further confirmed DWI-guided dose painting was an independent prognostic indicator of 2-year DFS and LRFS (*P* =.037 and *P* =.044, respectively). Detailed results of the multivariate analysis are presented in Table 4.

**Table 4:** Multivariable analysis of prognostic factors in locally advanced head and neck squamous cell carcinoma

Endpoint	HR (95% CI)	*P* value
Disease-free survival		
Sex	0.357 (0.145–0.880)	0.025
Age	1.209 (0.717–2.037)	0.477
T stage	0.300 (0.141–0.637)	0.002
N stage	0.435 (0.196–0.964)	0.040
Karnofsky scale	0.408 (0.191–0.874)	0.021
HPV status	0.923 (0.282–3.019)	0.894
Primary tumor site	1.265 (0.708–2.258)	0.427
DWI-guided dose painting	0.559 (0.324–0.966)	0.037
Local recurrence-free survival		
Sex	0.412 (0.156–1.088)	0.073
Age	1.280 (0.752–2.179)	0.364
T stage	0.310 (0.146–0.660)	0.002
N stage	0.457 (0.206–1.015)	0.054
Karnofsky scale	0.378 (0.175–0.814)	0.013
HPV status	0.877 (0.267–2.878)	0.829
Primary tumor site	1.186 (0.661–2.127)	0.568
DWI-guided dose painting	0.568 (0.328–0.984)	0.044
Regional recurrence-free survival		
Sex	3.344 (1.361–8.214)	0.006
Age	1.152 (0.640–2.075)	0.637
T stage	0.175 (0.062–0.491)	< 0.001
N stage	0.544 (0.242–1.223)	0.141
Karnofsky scale	0.505 (0.212–1.200)	0.122
HPV status	2. 334 (0.316–17.245)	0.406
Primary tumor site	1.217 (0.636–2.328)	0.553
DWI-guided dose painting	0.684 (0.378–1.238)	0.209
Locoregional recurrence-free survival		
Sex	0.331 (0.134–0.817)	0.017
Age	1.236 (0.731–2.089)	0.429
T stage	0.306 (0.144–0.650)	0.002
N stage	0.446 (0.201–0.988)	0.047
Karnofsky scale	0.394 (0.183–0.846)	0.017
HPV status	0.878 (0.268–2.877)	0.829
Primary tumor site	1.260 (0.704–2.256)	0.437
DWI-guided dose painting	0.558 (0.322–0.968)	0.038
Distant metastasis-free survival		
Sex	0.343 (0.129–0.916)	0.033
Age	1.372 (0.760–2.478)	0.294
T stage	0.307 (0.137–0.690)	0.004
N stage	0.450 (0.190–1.068)	0.070
Karnofsky scale	0.417 (0.182–0.955)	0.038
HPV status	1.066 (0.250–4.538)	0.931
Primary tumor site	1.208 (0.641–2.275)	0.559
DWI-guided dose painting	0.536 (0.293–0.979)	0.043
Overall survival		
Sex	0.525 (0.154–1.789)	0.303
Age	1.591 (0.785–3.223)	0.197
T stage	0.183 (0.055–0.606)	0.005
N stage	0.540 (0.206–1.414)	0.210
Karnofsky scale	0.398 (0.160–0.987)	0.047
HPV status	1.803 (0.239–13.598)	0.567
Primary tumor site	1.140 (0.533–2.441)	0.736
DWI-guided dose painting	0.885 (0.449–1.746)	0.725

Abbreviation: HPV = Human Papillomavirus; HR = hazard ratio; IC = induction chemotherapy;

## Discussion

HNSCC is notorious for its poor prognosis [26, 27]. Despite aggressive treatment strategies, Recurrence or distant metastases are the mostly reason for patients with LA-HNSCC make progressive. Therefore, patients with late-stage disease’ 5-year overall survival rate is about 50% [28]. IMRT has been widely used in HNSCC radiotherapy. This technique allows high-dose areas to closely conform to the tumor target volume, with the dose dropping sharply exterior these regions. However, accurately defining the tumor volume remains a key challenge [10]. Heterogeneity is an important source of variation in target volume selection and delineation of tumors. This heterogeneity can be quantified using ADC, which enhances LC. HNSCC is characterized by a high local recurrence rate. To combat drug resistance in LA-HNSCC, radiation dose escalation to the entire macroscopic tumor has been studied. Previous studies have indicated that an early increase in ADC values correlates with improved locoregional control (LRC) and suggests the potential to enhance local control by escalating the radiation dose to regions with low ADC values [29, 30]. Our findings underscore the role of dose escalation based on DWI-guided DP-IMRT in reducing local tumor recurrence.

DW-MRI offers superior functional ADC contrast and has demonstrated utility in evaluating treatment response and tumor-detecting NPC and HNSCC [9, 18, 31, 32]. This imaging modality enables precise delineation of the GTV, ensuring optimal dose delivery to the target volume while adhering to dose constraints for OARs. Our previous study [9] indicated that DWI-guided DP-IMRT confers a DFS benefit in patients with LA-NPC and does not exacerbate adverse events during short-term follow-up. Felice’s study [19] suggested that utilizing ADC-guided GTV delineation in the era of IMRT planning could be a safe approach to reduce the uncertainty in delineating tumor volume and, consequently, geographical misses. Lambrecht et al. [32] studied 175 patients with HNSCC and demonstrated that ADC values were prognostic factors for HNSCC recurrence. Similarly, Martens et al. [33] retrospectively included 134 patients with HNSCC treated with CCRT and identified DWI and 18 F-FDG-PET/CT parameters can be predictive to treatment failure, locoregional recurrence and death. These findings suggest that ADCmax-PT serves as a prognosis of treatment failure. Compared to positron emission tomography (PET)/CT, DW-MRI offers several advantages, including reduced medical costs, improved patient tolerance, and avoidance of radiation-related injuries. DW-MRI has been suggested as a predictive factor for tumor response to predict chemoradiotherapy [34]. Given these promising findings, we sought to evaluate the outcomes and toxicities of DWI-guided DP-IMRT compared to conventional MRI-based IMRT for the treatment of HNSCC.

In this study, the ADC < mean target was adopted as the clinical standard for dose escalation, consistent with previous clinical investigations wherein DP by contours was performed based on regions with ADC < mean [18]. ADC-based targets were the leading reason for dose distinctions. DWI-guided DP-IMRT, as an effective technique for dose escalation, can significantly increase the biologically effective dose delivered. Therefore, we chose to increase the biologically effective dose by about 10% in the DWI group compared to the MRI group. Adhering to the standard systemic regimen for LA-HNSCC, patients underwent chemoradiotherapy, with cumulative doses of > 200 mg/m^2^ generally recommended [35]. In our study, 98.4% and 97.8% of patients in groups DWI and conventional MRI, respectively, achieved this target dose. Most patients (99.2%) completed the planned RT dose. DWI-guided GTV with higher doses yielded complications similar to those in DWI group.

In a study by Montejoz, the CR rate after chemotherapy for LA HNSCC was 74% in Montejoz’s research [36]. In this study, the response rate in DWI group was favorable, with a higher CR rate in Group A (88.9%) compared to Standard group (77.6%). Vandecaveye et al.’s study [37] indicated that 2-year LRC and DFS were significantly correlated with the ΔADC values at 2 and 4 weeks. Another study, a phase II study [38] included 29 patients with late-stage LA-HNSCC receiving chemoradiotherapy, the 2-year progression-free survival (PFS) and OS rates were 71% and 75%, respectively. Michaelidou et al. [39] reported that the 1-year DFS rates were 83.3% and 78.9% at 3 years after PET/CT-guided IMRT. Patients in the DWI group in our study had a 2-year DFS rate of 75.2%. Our results suggest a reduction in local tumor recurrence with DWI-guided DP-IMRT.

As reported by Skorska et al. [40], acute toxicities did not increase with the DWI-guided DP-IMRT regimen compared to conventional MRI-based IMRT. The toxicity profile in our study was within the range of reported adverse events for LA-HNSCC treated with chemoradiotherapy [9, 10, 41, 42]. In DWI group, one patient developed a pharyngoesophageal stricture due to a hypopharyngeal primary lesion requiring a high dose to the lower pharyngeal constrictors and cervical esophageal inlet. Two patients had esophagitis because the primary tumor was located near the entrance of the esophagus. Approximately 11.9% of the patients in DWI group and 7.5% in Standard group had grade 3 dry mouth. The parotid glands’ mean dose was boosted to 39 Gy to the PTV in case of local tumor control. The incidence of Grade 3 oral mucositis was 59.5% in the DWI group and 66.4% in the standard group, with no significant difference between the two groups, which is consistent with the 53–83% incidence rate of grade acute mucositis reported in other studies [36, 43]. Some trials indicated that toxicities showed no significantly different between patients who were divided into standard chemoradiotherapy (70 Gy/35 fx) and dose escalation using dose painting (77 Gy/35 fx) groups, which is consistent with our study [42, 44]. In Welz’s study [42], acute toxicity levels reported in the high-dose and standard IMRT groups did not show significant differences.

Numerous studies have conveyed all kinds of prognostic factors for patients with HNSCC treated with CCRT. Radiation interruptions and delays during CCRT are known adverse prognostic factors [45]. In our study, none of the patients had treatment delays exceeding 5 days. The HPV status is a vital prognostic factor in patients with HNSCC. Eugenie’s study confirmed that the long-term OS for HPV-positive oropharyngeal squamous cell carcinoma is greatly improved compared to HPV-negative disease [46]. However, HPV status, a prognostic factor in head and neck cancer, was unknown in our study and could be a confounding factor.

Huang’s study [16] identified dose escalation as an independent prognostic factor for LRFS in patients with HNSCC. Law’s study [47] found that the skewness of the ADC distribution is an independent predictor of local recurrence in patients with NPC. Lambrecht’s prospective cohort study [32] reported that the ADC value is also an important prognostic factor in HNSCC, with a higher ADC most powerfully related to a worse prognosis. Our previous study showed that DWI-guided DP-IMRT was a prognostic factor for DFS and LRFS in patients with LA-NPC. To the best of our knowledge, no previous studies have explored the prognostic value of DWI-guided DP-IMRT in patients with HNSCC. Patil [48] et al.’s phase 3 trial reported that non-oropharyngeal sites of primary malignancy and stage III disease were associated with improved DFS. Our findings indicate that DWI-guided DP-IMRT is a significant predictor of DFS and LRFS in patients with HNSCC. Further prospective, randomized, controlled, large-sample studies on dose escalation for LA-HNSCC are needed.

## Limitations

This study had some limitations that should be acknowledged. Firstly, its retrospective nature limited the depth of analysis. Additionally, further follow-up was necessary to assess the long-term survival outcomes of patients with HNSCC. Therefore, sample sizes and prospective studies were required to prove these outcomes.

## Conclusion

DWI-guided DP-IMRT is associated with a DFS advantage without any discernible difference in toxicity among patients with LA-HNSCC.

## Data Availability

The original contributions presented in the study are included in the article. Further inquiries can be directed to the corresponding authors.
